# Food allergy in adults: the experience of a center in the north of Portugal

**DOI:** 10.1186/2045-7022-3-S3-P66

**Published:** 2013-07-25

**Authors:** M Couto, A Coimbra, D Silva, N Santos, A Pereira, JL Plácido

**Affiliations:** 1 Serviço de Imunoalergologia, Centro Hospitalar São João EPE; Immunology, Faculty of Medicine, University of Porto, Portugal; 2 Serviço de Imunoalergologia, Centro Hospitalar São João EPE, Porto, Portugal; 3 Serviço de Imunoalergologia, Centro Hospitalar São João EPE; Health Information and Decision Sciences Department Faculty of Medicine, University of Porto, Porto, Portugal

## Background

It is estimated that 2-3% of the adult population worldwide has 1 or more food allergies, but approximately 20% of the population avoids certain foods because of a perceived food allergy. We aim to describe the characteristics, implicated foods and final allergy diagnosis among adult patients with suspected adverse reactions to foods and to compare food allergic reactions (AR) and non-allergic reactions (NAR).

## Methods

A retrospective analysis of clinical files of patients followed in a University Hospital Allergy Department and referred to a Food Allergy Unit due to suspected adverse food reactions was undertaken. Adults studied between January 2009 and October 2012 were included. Demographic characteristics were recorded. Final diagnosis of food allergy was based on clinical history, sIgE, skin prick tests with commercial extracts and fresh foods, and open food challenges (OFC).

## Results

A total of 142 individuals (114 females; mean age 37±14y), with 236 suspected food adverse reactions, were included. Fifty six were confirmed as AR (24%, 43 patients) and 105 (45%) were considered NAR; in 75 (32%) the study was not concluded due to patient’s non attendance to the OFC. Anaphylaxis occurred more frequently and precociously (65% began<1h after food ingestion) in AR than in NAR (54% vs. 24%) while mucocutaneous symptoms were predominant in NAR (64%vs. 32%,p=0.002). In AR, the most implicated foods were crustaceans (36%), cephalopods (14%), fresh fruits (14%) and nuts (13%); in NAR, crustaceans (27%), fresh fruits (19%) and fish (14%). Patients with at least 1 AR had a higher prevalence of atopy (83% vs. 63%, p=0.027) and asthma (54% vs. 33%, p=0.037); rhinitis frequency was similar in both groups (96% in AR vs. 97% in NAR, p=0.799). Symptoms related to foods started mostly in adult age for both groups (mean age 27±14y for AR and 30±14y for NAR; p=0.17); 8 patients with at least 1AR, symptoms started in childhood (vs. 12 with NAR, p=0.880).

## Conclusion

Most of the adult’s adverse food reactions in our group were not allergic, however new allergies in adults should be studied since many food allergies begin in adult age. History of anaphylaxis should not presume an allergic etiology and OFC should be carried out. Implicated foods were similar between AR and NAR, and should not alter the investigation process. Non-adherence to the diagnostic work-up should be prevented by increasing patient awareness, avoiding unnecessary food restrictions.

## Disclosure of interest

None declared.

